# A Computerized Simulation of the Occlusal Surface in Equine Cheek Teeth: A Simplified Model

**DOI:** 10.3389/fvets.2021.789133

**Published:** 2022-01-03

**Authors:** Tomas Sterkenburgh, Ellen Schulz-Kornas, Michael Nowak, Carsten Staszyk

**Affiliations:** ^1^Veterinary Practice Dr. M. Nowak, Equine Clinic Meerbusch, Meerbusch, Germany; ^2^Department of Human Evolution, Max Planck Institute for Evolutionary Anthropology, Leipzig, Germany; ^3^Department of Cariology, Endodontology and Periodontology, University of Leipzig, Leipzig, Germany; ^4^Institute of Veterinary-Anatomy, Histology and Embryology, Justus-Liebig-University Gießen, Giessen, Germany

**Keywords:** horse, dentistry, dental wear, computer simulation, cheek tooth crown area, occlusal surface

## Abstract

Equine mastication, as well as dental wear patterns, is highly important for the development of treatments in equine dentistry. During the last decades, the stress and strain distributions of equine teeth have been successfully simulated using finite element analysis. Yet, to date, there is no simulation available for dental tooth wear in equines. In this study, we developed a simplified two-dimensional computer simulation of dental wear. It provides a first tentative explanation for the development of the marked physiological inclination of the occlusal surface and for pathological conditions such as sharp enamel points in equine cheek teeth. The mechanical properties of the dental structures as well as the movement of the mandible during the equine chewing cycle were simulated according to previously published data. The simulation setup was optimized in preliminary test runs. Further simulations were conducted varying the lateral excursion of the mandible and the presence or absence of incisor contact during the chewing cycle. The results of simulations showed clear analogies to tooth wear patterns in living equids, including the formation of wear abnormalities. Our analysis indicates that small variations in the pattern of movement during the masticatory cycle, as well as incisor contacts, are leading to marked changes in the occlusal tooth wear patterns. This opens new research avenues to better understand the development of dental wear abnormalities in equines and might have serious implications on captive animal health, welfare, and longevity.

## Introduction

For decades, the process of mastication as well as the principle of dental wear, in terms of attrition and/or abrasion, has been a matter of intensive research. Numerous scientific articles have elucidated the tribological mechanisms which explain the shape and function of dental surfaces in different herbivorous mammals (1–3). The occlusal surface of equine post canine dentition features a typical pattern of prominent enamel ridges, deep dentinal basins, and areas of dental cementum, altogether referred to as a “rasp facet” (4, 5). This morphology has been recognized as an effective adaptation to the herbivorous diet of modern equids (6). The enamel ridges function as shear-cutting edges while the dentine and cementum basins act as compression chambers (7–11). It is hypothesized that during the latero-medial-directed power stroke of the equine chewing cycle, the prominent enamel ridges slide over each other, shearing the forage that then becomes compressed in basins where cell contents are squeezed out (12). These functional adaptations of the rasp facet are not a primary feature of the equine dentition but are a result of a grinding process. When the equine tooth erupts into the oral cavity, the occlusal surface, rather than enamel ridges, is characterized by enamel cusps (covered by cementum). This primary occlusal surface has little to no functional relevance. The functional secondary occlusal surface (i.e., the rasp facet) forms as soon as antagonistic teeth contact each other, and the enamel cusps receive dental wear. As enamel is harder and more wear resistant than dentine and cementum (13, 14), it wears at a slower rate. This asynchronous wear rate between dental tissues produces the typical rasp facet morphology of prominent enamel ridges with neighboring regions of exposed dentine and cementum. Continued dental wear and compensating dental eruption ensure that these enamel ridges are sustained throughout the animal's life; this is often referred to as the “self-sharpening mechanism” (15).

While the functional and morphological aspects of the occlusal rasp facet of equine cheek teeth are well investigated, another remarkable characteristic of the equine post canine dentition—the lateroventral inclination of the occlusal surface—remains largely enigmatic. Although it is generally accepted that horse cheek teeth show an inclined occlusal surface under normal physiological conditions (16, 17), the definition of a functional healthy or pathological inclination is indistinct. It has been hypothesized that a less abrasion-dominated wear pattern could be linked to dental pathologies in captive equids, but to date, a link between reduced dietary abrasiveness and dental pathology has not been demonstrated (18). Previous research has shown that captive equids generally exhibit less abrasion-dominated tooth wear than their free-ranging conspecifics, and it was particularly evident that dental pathologies occur more in the premolar when compared to molar teeth [e.g., in focal overgrowths (hooks) of the rostral second premolars (106/206) according to the modified Triadan system (19)]. However, foundational morphological data and explanations on how these tooth pathologies are developed are currently missing.

Common approaches to investigate chewing motion analysis in mammals include usually highly precise but invasive animal experiments like 3D X-Ray Reconstruction of Moving Morphology (XROMM, (20)) or time-intensive feeding experiments (comparative feeding trails, (21)). In order to provide an alternative investigation option for equine dentistry as well as feeding experiments, we developed a simplified model to simulate the wear of occluding equine cheek teeth resulting in the occlusal surface inclination. We developed a model, since there is a rich record of published data available as basic input, e.g., of the occlusal surface geometry (4, 22), masticatory movements (23–26), and mechanical properties of dental material [enamel, dentine, cementum (13, 14, 27)]. Our aim is to simulate five exemplary wear cases using basic assumptions as simplistic wear approximations. These selected wear cases aim to image (a) occlusal wear during a life-long estimation as proof of concept and (b) test for a varying chewing motion pattern during increased mandibular buccal deflection.

## Materials and Methods

### Dental Abrasion

To describe loss of dental material in our simulations, we use the term dental abrasion (abrasio dentium) as a technical term referring to a loss of material caused by friction and mechanical wear (28, 29). Following basic principles of contact mechanics (30), we applied the following approximations:

- The volume loss V_abr_ due to wear is assumed to be proportional to the wear distance s_abr_ and the frictional force F_abr_ (Reye–Archard–Khrushchov wear law):
  $$\begin{array}{l} V_{abr}\;\sim \;s_{abr}\;*\;{\;F}_{abr} \end{array}$$
- The frictional force F_abr_ on the last molar (Triadan 11) should be about twice as high as on the second premolar (Triadan 06) due to the same torque and the shorter lever according to the law of levers and due to the bearing of the mandible on the temporomandibular joints and the muscular force action. At the same time, the lateral deflection due to a rotational movement in the TMJ on the last molar is about half that of the second premolar. We use a two-dimensional projection that does not consider these variations and assumes the friction force F_abr_ and the wear distance s_abr_ to be sufficiently constant over the entire arcade of the working side.

### A Model of the Equine Masticatory System

In a first step, we summarize morphological presumptions made on the masticatory process of domesticated horses that should underlie our model. These presumptions are as follows:

- The lateral width of the mandibular cheek teeth is assumed to be 2/3rd of the width of the maxillary cheek teeth (31). We define the symmetry position (centric occlusion) of the mandible during the chewing cycle as follows: the incisors are in contact; the mandible is positioned symmetrically to the median plane, and the interproximal space between teeth 101 and 201 (following the Triadan system of dental nomenclature) is aligned with the interproximal space between 301 and 401. While the resting position may vary from the symmetry position (centric occlusion), we assume both to be the same in our simulation.
- In the symmetry position, the slender mandibular cheek tooth rows show a medial overlap with the wider maxillary cheek tooth arcades of one third of the width of the maxillary cheek tooth arcades.
- In the symmetry position, the upper and lower cheek teeth are not in contact. During mastication, we assume that food particles keep the occluding teeth at minimal distance.
- As “chewing side,” we define the side on which the grinding process takes place. The contralateral “balancing side” is the side opposite the chewing side.
- The masticatory process is described by three phases (32):
  - The opening stroke: the jaw opens while the mandible moves laterally to the balancing side. When opening further, the mandible swings laterally back toward the median plane.
  - The closing stroke: while the mandible is moving laterally to the chewing side, the jaw closes until the buccal side of the lower cheek teeth is in a line with those of the upper cheek teeth and close to contact (assumption 1). However, the lateral deflection of the mandible at the end of the closing stroke may exceed the buccal aspect of the upper cheek teeth depending on the food material properties (assumption 2) (33).
  - The power stroke: the mandible moves back in lingual direction toward the symmetry position while the masticatory surfaces of the upper and lower cheek teeth of the chewing side slide over each other and grind the food in between. The power stroke ends close to the symmetry position, and a next chewing cycle starts with the following opening stroke (assumption 3). Rucker (34) describes a lateral bandwidth around the symmetry position, where the incisors get in touch causing a separation of the cheek teeth (assumption 4). We call this phenomenon the “incisor landing.”

### The Simulation Setup

The simulations were coded in the software language Processing (version 3.5.4), an open-source JAVA-based graphical framework with integrated development environment (www.processing.org) created by Casey Reas and Ben Fry under GNU Public License and GNU Lesser General Public License. The simulations were performed on a personal computer with the Intel Core I7 processor.

**Figure 1:**
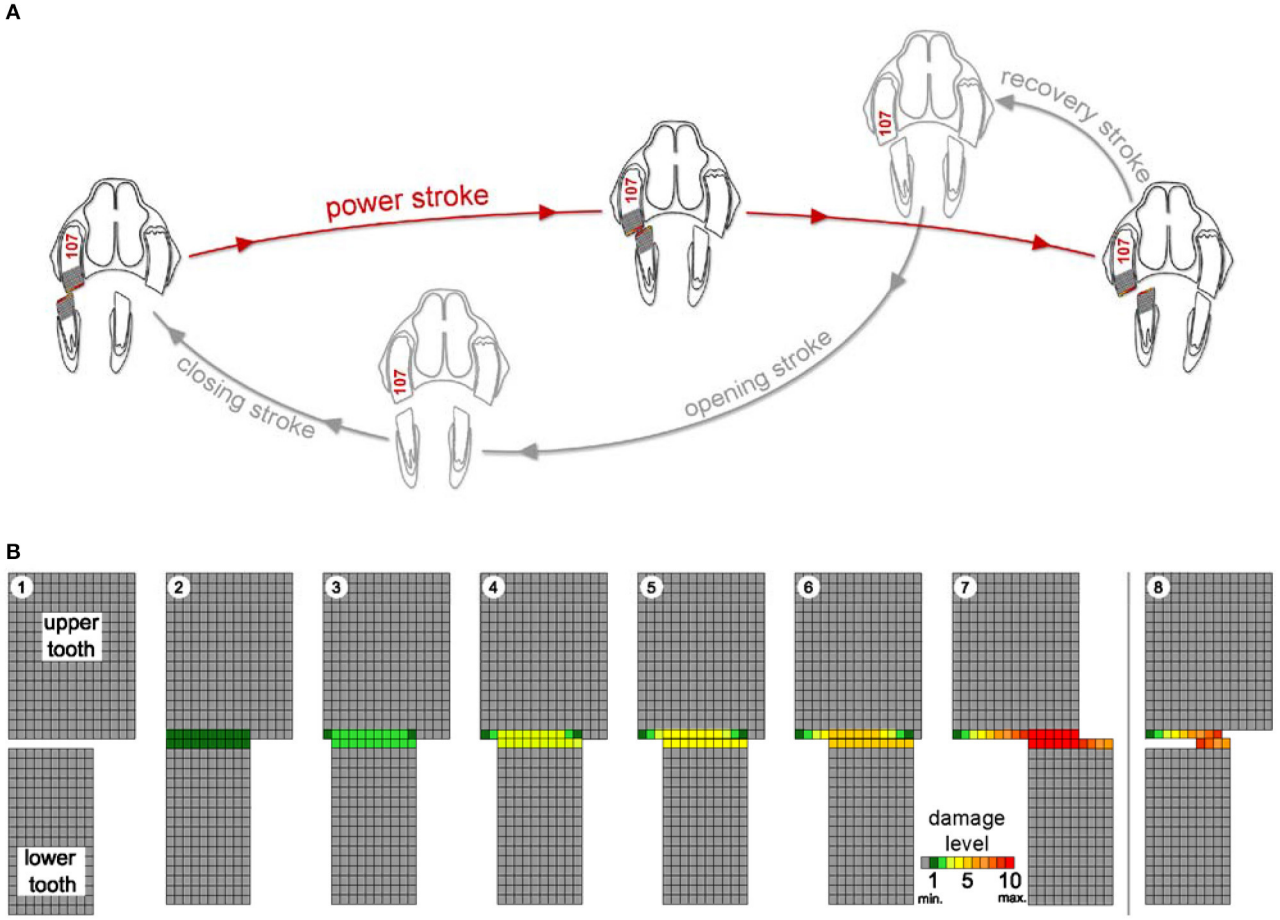
Schematic drawing of the chewing cycle **(A)** and a simulation of occlusal aspects of the teeth **(B)** illustrating the damage process as loss of pixel-by-pixel due to abrasional contacts. Upper and lower cheek teeth are represented as a two-dimensional rectangular matrix of a defined number of pixels. During the power stroke (steps 1 to 7), the lower cheek tooth moves in lingual direction across the upper cheek tooth pixel by pixel from left to right. Damage levels are given as number of touches per pixel (from minimum (min.) 0 = gray to maximum (max.) 10 = dark red). Step 8 indicates the starting point of the next power stroke. After every step, the damages of those pixels that are in contact with the antipode pixels are increased. When pixel damage exceeds the defined maximum damage value (10, dark red), it is erased (white).

**Figure 2:**
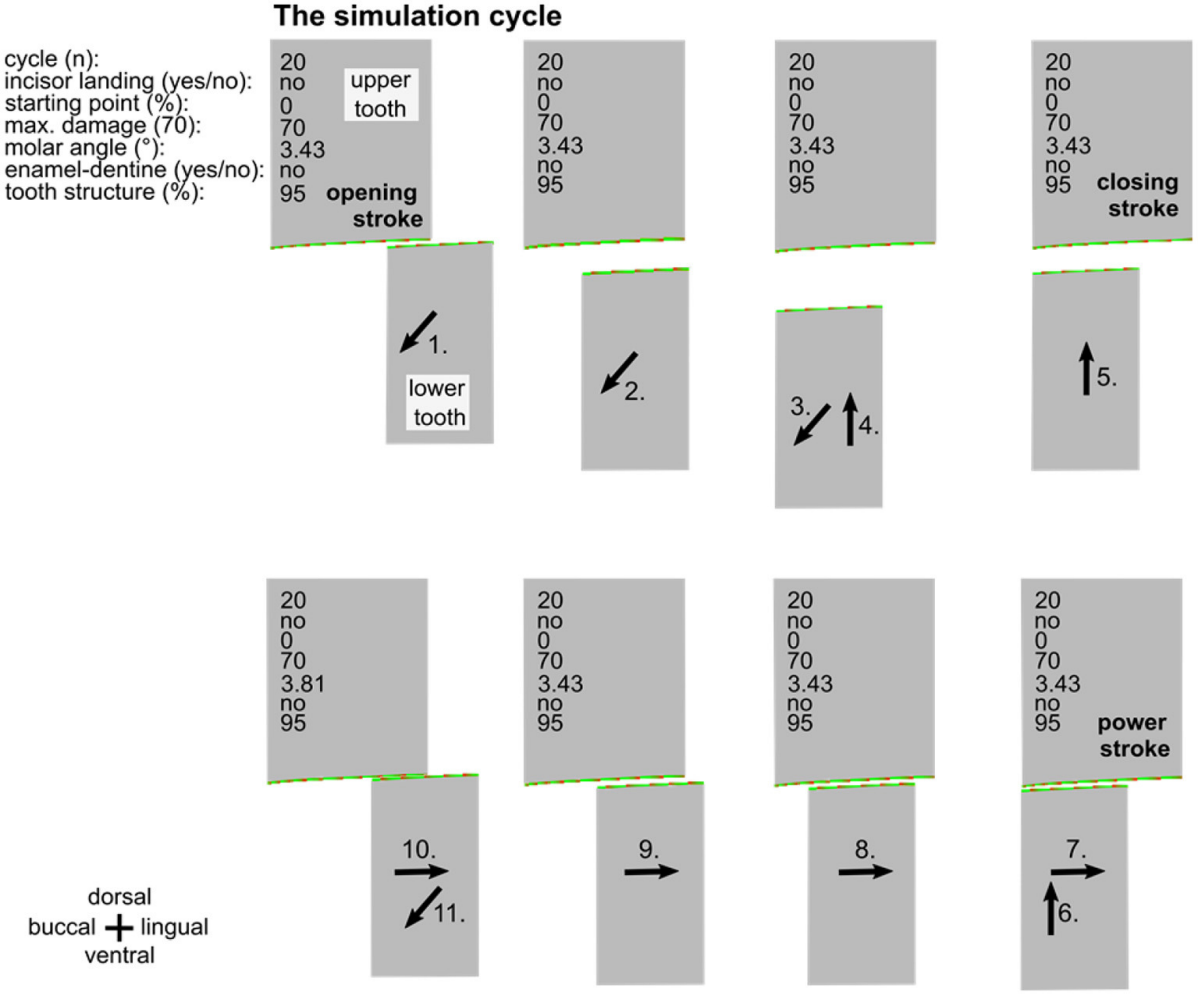
Overview of one entire simulation cycle (no. 20). The simulations consist of the opening stroke (1.-3.), the closing stroke (4.-6.), and the power stroke (7.-10), followed by a subsequent opening stroke (11.). Inscriptions specify the simulation parameters. Black arrows indicate the movement of the lower cheek tooth.

**Figure 3:**
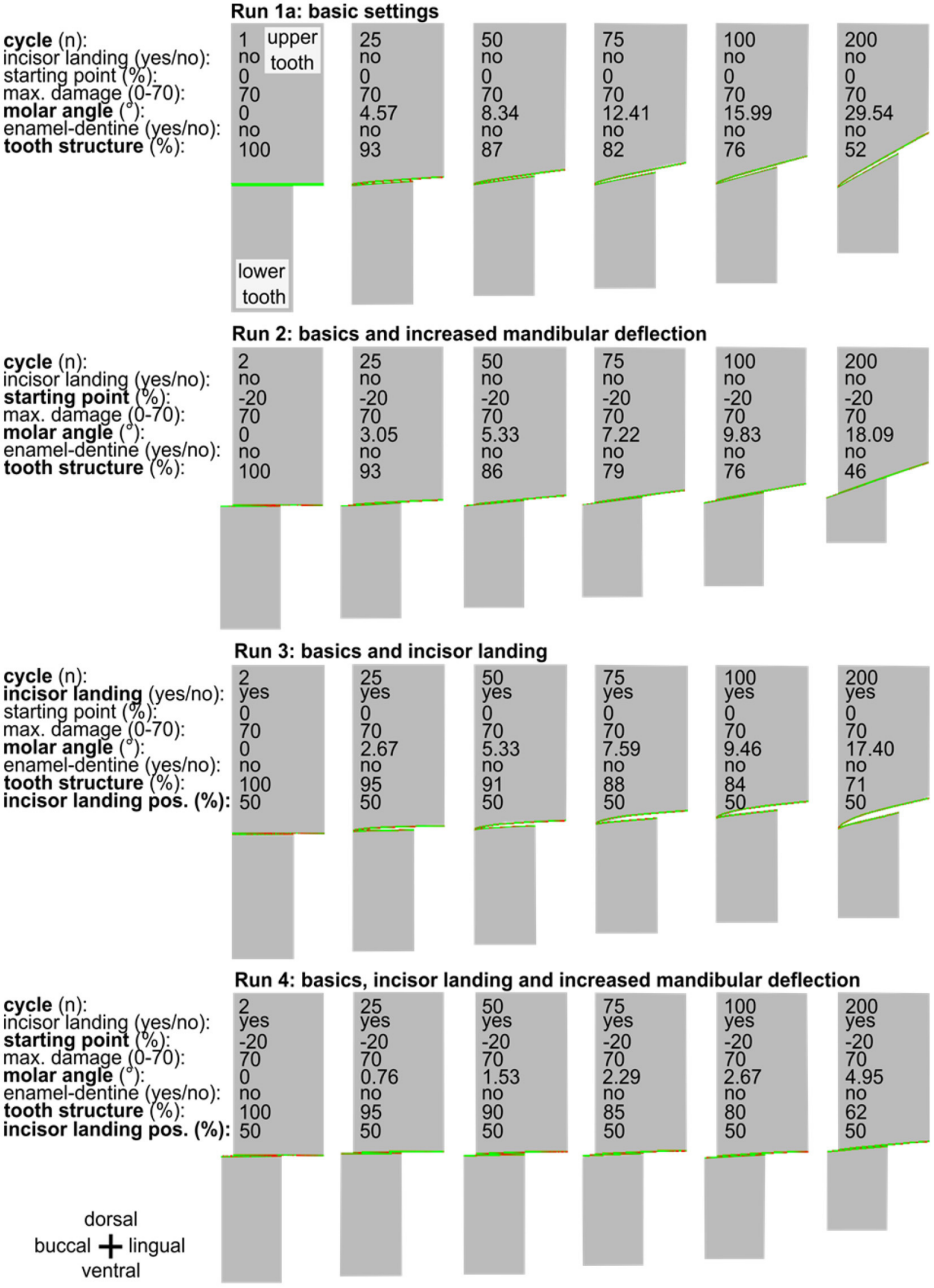
A series of simulation runs (1a to 4) displayed for two occluding cheek teeth varying the simulation settings (basics, incisor landing, and mandibular deflection). In each run from left to right, six selected points in time—each at the beginning of the power stroke—are presented for chewing cycle number 2, 25, 50, 75, 100, and 200. The inclination of the cheek teeth from buccal to lingual (left to right) is constantly increasing during the simulation, reaching various molar inclinations in cycle 200. Note the observation of a marked edge on the buccal side of the upper tooth in run 3.

Our simulation is meant to be a model and as such comes with a variety of simplifications and assumptions:

- The simulation is performed in two dimensions. It considers a two-dimensional cross section through the cheek tooth rows of the chewing side in the transversal plane. The simulation plane is thus spanned in the directions dorsal–ventral (top-bottom) and buccal–lingual/palatal (left-right).
- The tooth structure is considered to have a homogeneous wear resistance. We are well aware that the wear resistance of enamel, cement, and dentin is heterogeneous due to its different material properties; however, we aim to reach a higher degree of simplicity for the calculation and therefore start with a homogeneous wear resistance.
- The mastication cycle of the horse is simulated in the three phases (see (v) in the section above). As the opening stroke and the closing stroke in our model do not contribute to the tooth wear, we simplified the path of the mastication curve of those two phases to a linear movement. The course of the power stroke is determined by keeping the tooth–tooth contact of the worn teeth.
- The starting point of the power stroke (Figures 1, 2) is initially (Figure 3: run 1a and run 3) set in correspondence of the buccal tooth flanks. In additional simulations, we also considered an increased mandibular deflection (Figure 3: runs 2 and 4) and/or incisor landing (Figure 3: runs 3 and 4).
- We assume that during incisor landing the incisors carry the load of the masticatory muscle force together with the TMJs while the cheek teeth are not in contact and are running idle.

The simulation runs describe the loss of the cheek tooth structure for the upper and lower cheek teeth of the chewing side as an outcome of the chewing process. In a two-dimensional view, one upper cheek tooth and one lower cheek tooth are represented as a rectangular matrix of a defined number of pixels, as shown in the schematic drawing in Figure 1. This schematic drawing—for explanation purpose only—illustrates the core process of the power stroke simulation. The upper cheek tooth width is here set to 15 pixels, and the lower cheek tooth has a width of 10 pixels. Each pixel has a defined abrasion resistance. It is assumed that the masticatory force F_abr_ is constant over the full power stroke. Hereby, the volume loss due to abrasion can be considered as proportional to the friction distance s_abr_.

At the end of the closing stroke, the teeth are brought into contact at the initial position of the power stroke (Figure 1: B1). Then—during the power stroke—the teeth are moved across each other and become worn (Figure 1: B2 - B6) until the lower cheek tooth reaches the starting point of the opening stroke (Figure 1: B7). This is implemented by moving the lower cheek tooth pixel by pixel from left to right. After every step, the damages of those pixels that are in contact with the antipode pixels are increased. When pixel damage exceeds the defined maximum damage value (Figure 1: B7, red), it is erased (Figure 1: B8, white). When the pixels are erased, the underlying, undamaged next pixel becomes part of the new occlusal surface. Once this particular pixel gets into contact with an antipode pixel, the damage of this pixel is rising again until this pixel also exceeds the maximum damage and is erased as well. During the course of the simulation runs, this power stroke (Figure 1: B1–7) is part of every simulation cycle. It is performed repeatedly, and the loss of tooth structure forms a new shape of the occlusal surface.

A simulation run is created by repeated execution of masticatory cycles (Figure 2). In the simulation runs, the upper cheek tooth has a width of 150 pixels and the lower cheek tooth width is 100 pixels. The damage of the surface is also represented by colors from green (low damage) to red (high damage) while the maximum damage value is set to 70. By selecting this value, the amount of wear during the simulation equals approximately 6 years wear under physiological conditions in a horse. For every pixel that is in touch with the antipode pixel, the damage value is now increased by a random value between 0 and 2, taking into consideration that the damage is locally varying due to tooth structure hardness variations and due to the distribution of abrasives in the food.

## Results

The following simulation runs (except the longer run 1 b) consist of 200 mastication cycles. Input and output parameters are given in Table 1.

**Table 1:** Overview of selected simulation input and output parameters of the simulation runs.

**Simulation**	**Parameter description**	**Unit**	**Run 1a**	**Run 1b**	**Run 1c**	**Run 2**	**Run 3**	**Run 4**
Input	Starting point	%	0	0	0	−20	0	−20
	Incisor landing	None	No	No	No	No	Yes	Yes
	Angle at start	[°]	0	0	0	0	0	0
Output	Angle after 25 cycles	[°]	4.6			3.0	2.7	0.8
	Angle after 50 cycles	[°]	8.3			5.3	5.3	1.5
	Angle after 75 cycles	[°]	12.4			7.2	7.6	2.3
	Angle after 100 cycles	[°]	16.0			9.8	9.5	2.7
	Angle after 200 cycles	[°]	29.5			18.1	17.4	5.0
	Angle after 2.000 cycles			31.2				
	Angle after 20.000 cycles				31.2			
	Sharp enamel points	None	Minor	No	No	No	Yes	No

### Run 1a—Proof of Concept

The simulation run 1a shows that the characteristic angular inclination of the occlusal surface in the lateroventral direction became visible after 25 cycles and increased during further simulations (Figure 3). Loss of tooth structure and the abrasion is mainly defined by the selectable maximum damage value, which was set to 70 for a provisional simulation setup. Although this setup leads to an unrealistically high abrasion due to the low wear resistance of the tooth structure, it served as a check for a realistic outcome, at manageable simulation times of ~15 s per cycle.

### Run 1b and c—Long Runs

**Figure 4:**
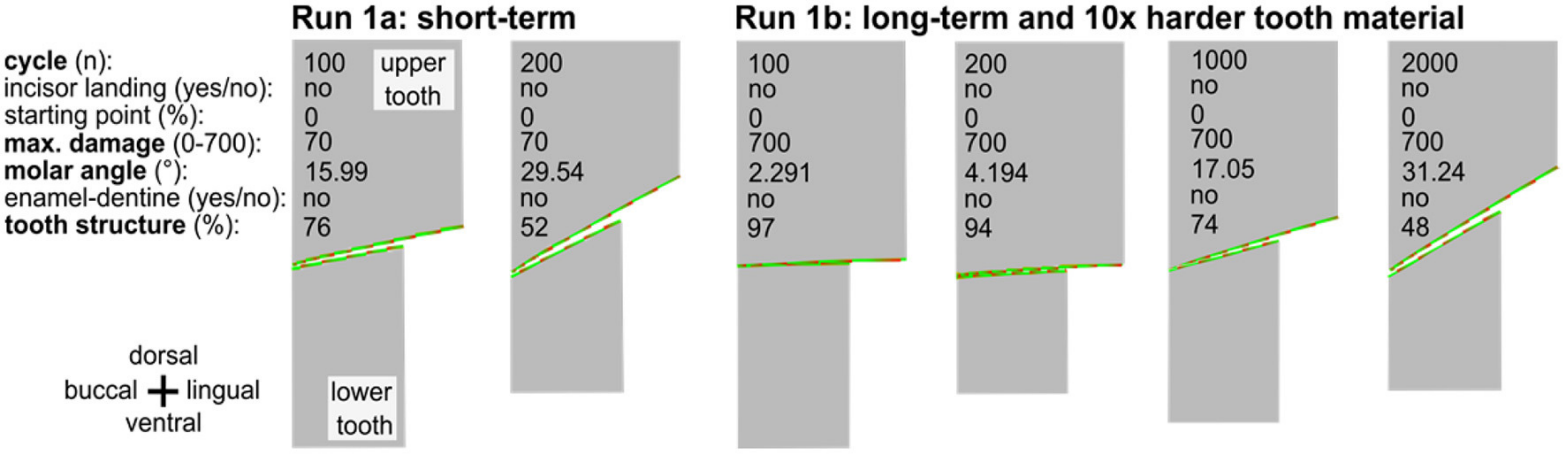
The long-term simulation run of an occluding upper and lower cheek teeth indicating 2,000 cycles with 10 × harder tooth material as compared to run 1a. The long-term run leads to very similar results after 1,000 or 2,000 cycles within the range of variation to be expected due to the random component in the tooth damage.

In the long run 1b, we tested a configuration with still unrealistic but tenfold and even hundredfold higher wear resistance and thus lower abrasion per cycle. In run 1c, we combined this change with a tenfold/hundredfold increase in the number of cycles, increasing the simulation time by a factor of 10/100 as well. In addition, the wear resistance was increased to 700/7,000. We compared the result after 1,000/10,000 and 2,000/20,000 mastication cycles with the results of run 1a after 100 and 200 cycles. All long-run simulations lead to similar results (divergence of <0.01%, see Table 1), taking into account the random parameters that are incorporated (Figure 4). We take this as an indication that simulations with realistic chewing cycle numbers in the range of 100 million within a horse's life and appropriately selected wear resistance should also lead to similar wear profiles as in our current simulation.

### Run 2—Increased Mandibular Buccal Deflection

Leue (35) and Bonin et al. (36) described that the mandibular deflection depends on the type of forage comminuted. Raw forage like hay seems to be associated with an increase in the mandibular deflection compared to softer food like oats. Taking this into consideration for run 2, we found that introducing raw forage leads to an increase in the mandibular buccal deflection of 20% of the mandibular cheek tooth width. This indicates that at the beginning of the power stroke the buccal flank of the mandibular cheek teeth reaches 20% further in the buccal direction (Figure 3). Simulating 200 chewing cycles again now led to a reduced cheek tooth angulation as compared to the run 1a (Figure 3).

### Run 3: Incisor Landing

In run 3 (Figure 3), we introduced a process that leads to an early termination of the power stroke due to incisor contact (incisor landing) which separates the cheek teeth.

This simulation run resulted in an edge that was formed on the buccal flank of the upper cheek tooth. We considered this edge to equal the phenomenon of sharp enamel points. At the same time, only a moderate angulation of 17.4° of the occlusal surface was formed.

### Run 4: Increased Mandibular Deflection in Combination With Incisor Landing

In the final run 4 of this series, we combined the increased mandibular deflection with the incisor landing (Figure 3). Here we observed the flattest angular inclination of 5° after 200 cycles and did not find any edge formation at the buccal/lingual flanks, i.e., no sharp enamel points were formed.

## Discussion

### The Proof of Concept (Run 1a and Run 1b)

As a result of run 1, we observe the emergence of the characteristic lateroventral inclination of the occlusal surface in cheek teeth. This effect can be explained by two mechanical facts: first, the occlusobuccal area of the upper cheek tooth is exposed to only little wear in the beginning of every power stroke. Second, the occlusobuccal side of the mandibular tooth undergoes the greatest possible wear over the entire power stroke, while its occlusolingual side is not in occlusion at the end of the power stroke and is thus exposed to less wear. Even though the direction of the inclination (lateroventral) can be explained with this simple model, the inclination angle becomes increasingly steep over the course of the simulation and appears to exceed the physiological level of ~15° (34). The observation of continuously increasing occlusal angles within the simulations is in contrast to the *in vivo* situation observed in horses. Although wide interindividual and intraindividual ranges are reported (17), the occlusal angles remain more or less constant in an individual horse under physiological conditions. Therefore, we assume that horses possess an optimal angulation and are able to preserve this optimized condition, presumably by adjusting their mandibular movements during the masticatory action. Thus, the equine dentition provides an enormous morphological plasticity to maintain functionality; e.g., adjusting to variation in lifelong dental wear and tooth eruption. This ability of equines to make continued adjustment of cheek tooth angulation, in the face of continued wear and eruption, indicates a reliable, self-adjusting mechanism to guarantee a lifelong optimized occlusal surface geometry and thus effective food comminution. Such self-adjustment requires the equine masticatory apparatus to have high sensory feedback and fine motor control, which has been already documented for human and rodents, e.g., rats and mice (27). In horses, the complexity of the afferent and efferent innervation of the masticatory apparatus might even exceed that of non-hypsodont mammals; however, more research is needed to solidify this assertation.

Simulating the amount of tooth wear required an estimation of material loss and chewing duration. A minimal wear of tooth material in equids is estimated with a value of 2 mm at the occlusal surface (37) and minimal 5 h of chewing are assumed daily with about one chewing cycle per second (38). It would suggest a need of more than 3 million power strokes to grind down 1 mm of dental material (see formula below).


$$\begin{array}{l} 1\frac{\text{cycle}}{\sec}\;*\;3600\;\frac{\sec}{\text{h}}*\;5\;\frac{h}{d}\;*\;365\;\frac{d}{y}\;*\;\frac{0,5y}{mm}\sim 3.3Mio\;\frac{cycles}{mm} \end{array}$$

with sec = seconds, d = days, h = hours, y = years, mm = millimeters

Thus, in a horse, the estimated minimal loss of tooth material (wear) of the cheek teeth is in the order of several millions of chewing cycles per mm of wear. Here, we want to emphasize that our simulations are first approximations with some limits and simplifications. We are aware that, in order to simulate the life span of a horse (up to 30 years), this simulation would necessitate several hundred million cycles. In order to accommodate a “life span,” simulation would require a substantial increase in computing powerful computers and optimization of the current programming. Yet, with our setup we needed ~15 s per chewing cycle. However, in run 1b we performed a simulation with a ten/hundred-times higher tooth wear resistance, which lead to similar results after ten/hundred-times more chewing cycles. The amount of wear during the simulation equals ~6 years under physiological conditions in a horse. Nevertheless, the obtained results imply that our very simplistic simulations reproduce the general wear pattern found in equids. We suppose that it can be used as an approximation and proof of concept for tooth wear pattern in equids testing various occlusal situations.

### Increased Mandibular Buccal Deflection and Incisor Landing (Run 2–4)

The implementation of two variations of the mandibular movements (larger buccal deflection, incisor landing) produced significant alterations of the simulated outcomes. A larger deflection of the mandible during the closing stroke leads to a slower increase in the occlusal angulation and thus results in a flatter angulation after a given number of power strokes. However, it remains unclear whether an enlarged mandibular deflection in the living horse just results in a slower increase in the angulation or whether this condition establishes a constant flatter angulation. Nevertheless, as it has been demonstrated that mandibular deflection increases with particle size of the forage (36), and therefore, it is assumed that diet has a significant influence on the adjustment of the occlusal cheek tooth angulation in horses. Consequently, changes in diet should lead to a readjustment of the preferred occlusal angles. Thus, the reported wide ranges of equine cheek teeth angulations (17) might be—at least in part—explained by variations of particle sizes of the forage.

The obtained simulation outcomes suggest an accelerated increase in the angulation with minimized mandibular deflection which may cause an extreme occlusal angulation. Such a condition is observed in horses showing so-called shear mouth. Although several different definitions of the term shear mouth exist (39), it is generally accepted that an abnormal steep angulation of the cheek teeth is the main characteristic of this pathological condition. It has been shown that shear mouth develops in cheek tooth rows with ipsilateral, painful dental diseases (39). This observation strongly suggests that horses do not use the affected cheek tooth rows as the working side during the chewing process but solely as the balancing side to minimize pain sensation. Currently, the development of the excessive occlusal angulations in shear mouth was explained by a partial lack of wear on the buccal margin of maxillary cheek teeth and the lingual margins of the mandibular cheek teeth (37, 40, 41). However, cheek teeth in shear mouth show completely worn and exceedingly slanted surfaces rather than a partly worn surface with some dental overgrowth. According to our model, shear mouth geometry results from complete grinding of the total occlusal surfaces by jaw movements with minimized lateral deflections rather than by incomplete grinding of the occlusal surfaces and resulting partial dental overgrowth. However, further studies are required to confirm the results obtained by computerized simulations with data from clinical investigations.

In clinical examination, horses can usually perform a lateral shift of the mandible for a few mm out of the neutral position while incisors remain in contact. Confirmed by endoscopic examination (42), the cheek tooth rows levitate without tooth–tooth contact in that phase of lateral shifting. As soon as the antagonistic cheek teeth reach contact, further lateral shifting causes an opening of the mouth, as the mandibular cheek teeth are guided in the lateroventral direction due to the angulated occlusal surfaces, and thus, the incisor arcades lose contact. This phenomenon is widely used in clinical practice as pre-assessment for the indirect determination of the inclination angle of the occlusal surface of the cheek teeth (34). The described correlation of incisor contact and lateral shift of the cheek tooth rows suggests that incisor arcades reach contact in the final phase of the power stroke. Consequently, we modified our simulations accordingly and implemented an incisor contact causing a separation of the cheek teeth at the end of the power stroke, referred to as “incisor landing.”

Interestingly, the incisor landing combined with a non-increased buccal deflection protruding edges on the occlusobuccal rim of the maxillary cheek tooth produced results that resemble the clinically relevant phenomenon of sharp enamel points. However, protruding edges on the occlusolingual rim of the corresponding mandibular cheek tooth were not produced by the present simulation, which is in contrast to the typical clinical finding that sharp enamel points usually occur concomitantly in mandibular as well as in maxillary cheek teeth. Therefore, it remains unclear whether the conditions used in the present simulation reflect the situation in live horses appropriately. Nevertheless, it has been shown that our model provides an experimental tool to simulate the development of structures resembling sharp enamel points in equine cheek teeth and, thus, enables the identification of the relevant factors contributing to this dental malformation. In order to improve the reliability of further simulations, the assessment and implementation of clinical and anatomical data are urgently required. In addition, the potential use of 3D fluoroscopy could help to more accurately describe the motion of the mandible as a preparatory tool for more refined computer models or, e.g., XROMM has been used to evaluate mastication in juvenile pigs (43).

## Conclusion

We proposed a simulation of the equine chewing process, based on a highly simplified model of the masticatory process. It considers a two-dimensional model of upper and lower cheek teeth of the chewing side. In four runs, we simulated basic scenarios that lead to healthy as well as to pathological dental wear through chewing. Simulation outcomes allowed the formulation of biomechanical explanations for the transition of the non-tilted primary occlusal surface into the functional, lateroventrally angulated secondary occlusal surface. An extended buccal deflection at the beginning of the power stroke was identified as a significant factor decreasing the speed of continued inclination. However, as inclination steadily increased during simulations instead of approaching a constant final value, we propose that optimal cheek tooth angulation becomes adjusted in living horses by adapted chewing movements which stabilize an optimal and convenient angulation. Thus, in addition to a self-sharpening mechanism as proposed by Dixon (44), equine cheek teeth might possess a self-adjusting mechanism too.

Surprisingly, the implementation of an incisor landing led to simulation outcomes resembling sharp enamel points. However, we are aware that these simulations reflect the *in vivo* situation in a first approximation only. This finding indicates that the presented model has the potential to elucidate factors causing typical pathological dental changes in the equine dentition. However, further refinements of the model and comparison of simulation outcomes with *in vivo* morphologies are required in order to establish a tool which allows simulating the etiopathological process as well as proposed treatment options in a reliable way.

## Data Availability Statement

The original contributions presented in the study are included in the article/Supplementary Materials, further inquiries can be directed to the corresponding authors.

## Author Contributions

TS and MN designed the study. All authors contributed to the analysis, interpretation, and improvements. TS conducted the simulation and drafted the manuscript. All co-authors wrote the paper.

## Conflict of Interest

The authors declare that the research was conducted in the absence of any commercial or financial relationships that could be construed as a potential conflict of interest.

## Publisher's Note

All claims expressed in this article are solely those of the authors and do not necessarily represent those of their affiliated organizations, or those of the publisher, the editors and the reviewers. Any product that may be evaluated in this article, or claim that may be made by its manufacturer, is not guaranteed or endorsed by the publisher.

## Supplementary Material

Supplementary Video 1 Video of the simulation run 3 using the following simulation settings: normal simulation speed over cycle 1-3, cycle 4-10 with 4x time-lapse, cycle 11-94 with 32x time-lapse, cycle 95-105 with 4x time lapse, cycle 106-198 with 32x time lapse, cycle 198-200 without time-lapse: run 2.mp4.

Supplementary Material 1 Markdown file of the software program code: Markdown.pdf.
