# Supplementary material for: A Computerized Simulation of the Occlusal Surface in Equine Cheek Teeth: A Simplified Model
Source: Front Vet Sci. 2022 Jan 3;8:789133. doi: 10.3389/fvets.2021.789133 (PMC8761981; doi:10.3389/fvets.2021.789133)
Supplement: Supplementary Material 1 — Markdown file of the software program code: Markdown.pdf. [file Data_Sheet_1.PDF]

1. Markdown

1.1 General Information

The program consists of 4 main components. The setup() – procedure is processed once at the beginning of the simulation and takes care for most of the initialization. The draw() - procedure is performed once with every movement step of the mandibular cheek tooth and takes care for:

(i) Printing the text output,
(ii) Rendering the teeth
(iii) Moving the teeth during the 3 strokes and calculating the damage during the power stroke
(iv) Counting the simulation cycles

The teeth are handled as objects of the class Tooth().

1.2 The Class Tooth()

The class Tooth owns two PVector objects, a position vector pos and a movement vector move

```
class Tooth {
  PVector pos;
  PVector move;
```

The central array, keeping the tooth structure alive over the elements along the toothWidth is the array toothStructure[][]

```
  int[][] toothStructure;
```

It consists of 3 layers, selected by the second dimension: "0" to store the tooth surface dimension in dorso-ventral direction, "1" to store the damage value, "2" to store the structure preset, equaling the tooth hardness.

```
final int YVALUE = 0;
final int DAMAGE = 1;
final int STRUCTURE_PRESET = 2;
```

The following parameters may be adjusted:

```
final int max_DAMAGE = 70;
final boolean toothBodyVisualization = true;
final boolean SIMULATE_ENAMEL_DENTINE = false;
```

The maximum damage value representing the maximum tooth hardness. Defining, if the full tooth or only the contact line should be rendered. Defining, whether a hardness profile should be loaded.

```
final float[] structure = {  6.2, 8, 8.8, 10.4, 13.2, 14.6, 15, 15.2, 14, 11.6,
                            11.8, 12.4, 13.6, 13.6, 12.8, 11.6, 11, 9.2, 9, 9.2,
                            9.2, 9.2, 9, 8.6, 9.4, 9.2, 9, 8.8, 9, 8.4, 9, 8,
                            7.4, 6.4, 5.6, 5, 5.6, 5.8, 5.2, 4.2};
```

The tooth hardness profile may be loaded as an array of hardness values as a buccal to lingual hardness profile; averaged in rostro caudal direction. This option is experimental, still.

The tooth comes with further properties:

```
int toothWidth;
int heightMolar;
boolean maxilla;
int colorChannel;
```

The toothWidth and heightMolar defines the width and height of the tooth in pixels. Maxilla is true for the maxilliar tooth, false for mandibular tooth. colorChannel is needed for rendering purposes, only.

The tooth constructor comes with the parameters position, width, height of the tooth (heightMolar) and maxilla and is performing the following:

Copying the parameters to internal variables of the same name to keep them alive.

Creating the toothStructure -Array with the 3 layers for tooth length, damage value and structure preset.

Creating the PVector “move” and setting it to (0,0) for x and y direction, initializing the YValue and the damage to 0, filling the structure preset, if ENAMEL_DENTINE simulation is chosen or setting it to a constant max_damage, if not. toothStructure[i][STRUCTURE_PRESET] defines the maximum damage value for every pixel over the width of the tooth, because it is still experimental: with a somewhat unconventional calibration.

```
Tooth(PVector pos, int toothWidth, int heightMolar, boolean maxilla ) {

  this.pos = pos;
  this.toothWidth = toothWidth;
  this.maxilla = maxilla;
  this.heightMolar = heightMolar;
  toothStructure = new int[toothWidth][3];
  move = new PVector(0, 0);
  for (int i = 0; i <toothWidth; i++) {
   toothStructure[i][YVALUE] = 0;
    toothStructure[i][DAMAGE] = 0 ;
    if (SIMULATE_ENAMEL_DENTINE) {
      toothStructure[i][STRUCTURE_PRESET] = int(structure[i*40/toothWidth]*MAX_DAMAGE/15.2);
    } else {
      toothStructure[i][STRUCTURE_PRESET] = MAX_DAMAGE;
    }
  }
}
```

Rendering the teeth is done with the method render(). It starts with a pushMatrix() and ends with a popMatrix() in order to protect transformation, performed outside this function. In a next step the object is moved to a central position in the window by a translate(). The colorChannel is used to define the color representing the damage of the tooth. It is scaled for color values between 0 and 255 and is used for the RGB definition from green to red in later drawing procedures utilizing stroke(R,G,B).

colorValue defines the color of the tooth body and is scaled between black and yellow, depending on the tooth hardness. stroke(colorValue, colorValue,0) is setting the appropriate yellow tone.

The tooth body is built out of vertical lines of one pixel in width. In order to draw the line correctly, the procedure destinguishes between maxilla and mandibula. The yellow lines, representing the

tooth body are only drawn, if toothBodyVisualization is true. For every i iterating along the tooth width the line for the maxilla is drawn vertically in the x-position pos.x+i from the starting position pos.y to the length of the tooth at the specific x-position, given by pos.y + heightMolar – number of abrased pixels, given by toothStructure[i][YVALUE]. This is zero in the beginning and will increase with every abrased pixel through the draw() routine during the simulation process. For the mandibula arcade the „else“ comes into play and the drawing shows the abrased pixels on the other end of the tooth.

```
  void render() {

    pushMatrix();
    translate(width/3-50, height*2/3-275);
    for (int i = 0; i < toothWidth; i++) {
      colorChannel = 255 - 250*toothStructure[i][DAMAGE] / MAX_DAMAGE;
      int colorValue = toothStructure[i][STRUCTURE_PRESET]* 180 / MAX_DAMAGE;

      stroke(colorValue, colorValue, 0);
      if (maxilla) {
        if (toothBodyVisualization) {
          line(pos.x + i, pos.y, pos.x + i, pos.y + heightMolar - toothStructure[i][YVALUE]);
        }
        stroke(255 - colorChannel, colorChannel, 0);
        line(pos.x + i, pos.y + heightMolar - toothStructure[i][YVALUE],
             pos.x + i, pos.y +  heightMolar - 2 - toothStructure[i][YVALUE]);

      } else {

        if (toothBodyVisualization) {
          line(pos.x + i, pos.y + heightMolar, pos.x + i, pos.y + 2 + toothStructure[i][YVALUE]);
        }
        stroke(255 - colorChannel, colorChannel, 0);
        line(pos.x + i,  pos.y + 2 + toothStructure[i][YVALUE],
             pos.x + i,pos.y + toothStructure[i][YVALUE]);
      }
    }
    popMatrix();
  }
}
```

Finally the damage visualization takes place. As one pixel is difficult to see, a short, vertical 3 pixel line is drawn in a color between green and red. The color is set by stroke(255-colorChannel, colorChannel, 0), increasing RED and reducing GREEN with every step.

1.3 The main program

1.3.1 Definition of the various strokes

In a first step the main program defines constant values representing the various strokes and setting the stroke variable to POWERSTROKE

```
final int POWERSTROKE    = 1;
final int OPENINGSTROKE  = 2;
final int CLOSINGSTROKE  = 3;
final int INCISORTOUCH   = 4;

int stroke = POWERSTROKE;
```

1.3.2 Calculating the contact line

As abrasion will happen on the contact line, variables for the starting point and the end point of the contact line are defined:

```
int startContactLine, endContactLine;
int neutral_position_x;

int i_Lower;
int delta_pos_x;
```

1.3.3 Parameterization of the Simulation

The following variables parameterize the simulation. The width of the lower and the upper molar, the height of the molars, the starting point for the power stroke. For our runs we used either a zero for buccal correspondence or 1/5th - equaling 20% - of the width of the lower molar.

Incisor landing may be set to true or false and the landing point is defined as incisor_touch_point.

stopAtRemaining defines the end of the simulation run at a defined percentage (here 30%) of abrased tooth material at the right flank of the lower molar.

```
final int WIDTH_UPPER_MOLAR  = 150;
final int WIDTH_LOWER_MOLAR = 100;
final int HEIGHT_MOLAR    = 200;



int powerStrokeStartPoint = 0;
// int powerStrokeStartPoint = - int(WIDTH_LOWER_MOLAR/5.0);


final boolean SIMULATE_INCISOR_LANDING = true;
final float incisor_touch_StartPoint = 0.50;
final int stopAtRemaining = 30;
```

1.3.4 Tooth() objects and initialization

Two objects of the class Tooth are needed for the simulation run. Cycle is the number of the simulation cycle and in touch defines whether or not the teeth are in touch.

```
Tooth upperTooth, lowerTooth;
boolean in_touch;
int cycle;
```

The setup() procedure sets the frame for the simulation output to 300 times 750 pixels, calls the initSimulation and sets the color mode to a 256 step RGB.

```
void setup() {
  size(300, 750);
  initSimulation();
  colorMode(RGB, 255);
}
```

initSimulation is creating the Tooth objects upper_Tooth and lower_Tooth, is calculating the neutral_position. The move vector is initialized to one step in positive x-direction and the cycle is set to 1.

```
void initSimulation() {
  upperTooth = new Tooth(new PVector(0, 0), WIDTH_UPPER_MOLAR, HEIGHT_MOLAR, true);
  lowerTooth = new Tooth(new PVector(0, HEIGHT_MOLAR), WIDTH_LOWER_MOLAR, HEIGHT_MOLAR, false);
  neutral_position_x = upperTooth.toothWidth
                      - int(float(lowerTooth.toothWidth) * incisor_touch_StartPoint);
  lowerTooth.move.x = 1;
  lowerTooth.move.y = 0;
  cycle=1;
}
```

1.3.5 Text Output

textOutput() is taking care for the text output in the simulation window. The first part is straight forward.

```
void textOutput(){

      pushMatrix();
      translate(20, 20);
      textSize(16);
      fill(60);
      switch (stroke){
        case POWERSTROKE:
            text("Power Stroke", 10, 30);
          break;
        case CLOSINGSTROKE:
          text("Closing Stroke", 10, 30);
          break;
        case OPENINGSTROKE:
          text("Opening Stroke", 10, 30);
          break;
        case INCISORTOUCH:
          text("Incisors landed", 10, 30);
          break;
      }
      text("cycle No.: ", 10,50);
      text(cycle, 200,50);
      text("incisor landing:", 10,70);
      if (SIMULATE_INCISOR_LANDING){
        text("yes", 200,70);
      }
      else{
         text("no ", 200,70);
      }
      text("starting point:",10,90);
      text(powerStrokeStartPoint, 200,90);
      text("max. damage",10,110);
      text(lowerTooth.MAX_DAMAGE, 200,110);
      [illegible]
```

In the definition of the tangent as the opposite cathetus through the adjacent cathetus, the angle can be determined as the arcus tangent using the corresponding parameters, measured at the upper molar. The remaining part of textOutput() is straight forward again.

```
      text("molar angle:",10,130);
      text(degrees(atan(float(upperTooth.toothStructure[WIDTH_UPPER_MOLAR-1][YVALUE]
      - upperTooth.toothStructure[0][YVALUE])/float(WIDTH_UPPER_MOLAR))), 200,130);
      text("enamel-dentine sim.",10,150);
      if (upperTooth.SIMULATE_ENAMEL_DENTINE){
        text("yes", 200,150);
      }
      else{
         text("no ", 200,150);
      }
      int toothRemaining = 100-100*lowerTooth.toothStructure[0][0]/HEIGHT_MOLAR;
      text("rem. tooth struct. (%):",10,170);
      text(toothRemaining, 200,170);
      if (toothRemaining <= stopAtRemaining){noLoop();}
      if (SIMULATE_INCISOR_LANDING == true){
        text("Incisor land. pos. (%):",10, 190);
        text(int(incisor_touch_StartPoint*100) , 200,190);
      }


      popMatrix();
}
```

1.3.6 Rendering the teeth

The central draw() routine starts with setting the background color, doing the textOutput() and rendering both teeth.

```
void draw(){

  background(240);
  noFill();
  textOutput();
  lowerTooth.render();
  upperTooth.render();
```

1.3.7 A State machine handling the strokes

The change between the various strokes is initiated by reaching certain positions. At those positions the move – vector is changing and the movement follows the new direction. The first if clause checks for the end of the power stroke. If reached, the stroke changes to opening stroke, the movement goes one step buccally and one step ventrally at the same time. The “cycle”- variable is increased by one at that point.

When the buccal flank of the lower tooth reaches the lateral position of the powerStrokeStartPoint, the closing stroke is initiated. The lower tooth keeps the x-position and moves in dorsal direction until both teeth are in touch and the power stroke starts again. If SIMULATE_INCISOR_LANDING is true, there is a small fourth, additional period at the end of the power stroke, when the cheek teeth run idle and the mandibula moves laterally towards the starting point of the opening stroke. This phase is named INCISORTOUCH.

The move vector during the power stroke is handled later in this document.

With the start of the power stroke a saveFrame() saved the pictures, used in this paper at cycle number 2, 25, 50, 75, 100 and 200.

```
    if ((lowerTooth.pos.x > neutral_position_x) &&
        ((stroke == INCISORTOUCH) || (SIMULATE_INCISOR_LANDING==false))){
      stroke = OPENINGSTROKE;
      cycle++;
      lowerTooth.move.x = -1;
      lowerTooth.move.y = 1;
    }
    if ((lowerTooth.pos.x <= powerStrokeStartPoint)
         && (lowerTooth.pos.y > HEIGHT_MOLAR + 15
                                  - upperTooth.toothStructure[1][YVALUE]
                                  - lowerTooth.toothStructure[1][YVALUE])){
      stroke = CLOSINGSTROKE;
      lowerTooth.move.y = -1;
      lowerTooth.move.x = 0;
    }
    if ((lowerTooth.pos.x <= powerStrokeStartPoint)
             && (lowerTooth.pos.y <= HEIGHT_MOLAR)){
      stroke = POWERSTROKE;
      if ((cycle == 2)||(cycle == 25)||(cycle == 50)||
          (cycle == 75)||(cycle == 100)||(cycle == 200)){
        saveFrame("sim_" + cycle + "_" + abs(powerStrokeStartPoint) +
        "_" + SIMULATE_INCISOR_LANDING + ".png");
      }
    }
    if ((lowerTooth.pos.x > upperTooth.toothWidth * incisor_touch_StartPoint)
         && (stroke == POWERSTROKE)&& SIMULATE_INCISOR_LANDING){
      stroke = INCISORTOUCH;
      lowerTooth.move.x = 1;
      lowerTooth.move.y = 0;
    }
```

1.3.8 The Power Stroke

While the other strokes are only designed to move the lower cheek tooth in a defined direction, the power stroke handles the abrasion process.

The Power stroke is initiated, when the condition for reaching the starting point in x- and y- direction is met. At that point the stroke is set to POWERSTROKE.

During the power stroke the movement pattern is more complex. This is because over the time the contour of the tooth is changing. In order to identify those pixels per x-position of the moving mandibular tooth that are in contact we apply the following procedure:

The variable in_touch is defined to monitor whether or not there is tooth-tooth contact. In the beginning it is set to false. The lower mandibular tooth is moving in a pattern, which goes one step lingually in x-direction and two steps ventrally in y-direction.

The overlap of both teeth in x-direction is identified to start at startContactLine and ending at endContactLine. The delta in x-direction between both teeth positions is calculated and stored as delta_pos_x.

```
if (stroke == POWERSTROKE){
    lowerTooth.move.x = 1;
    lowerTooth.move.y = 2;
    in_touch = false;
    startContactLine = max(int(upperTooth.pos.x), int(lowerTooth.pos.x));
    endContactLine  = min(int(lowerTooth.pos.x) + lowerTooth.toothWidth,
                          int(upperTooth.pos.x) + upperTooth.toothWidth);
    delta_pos_x= int(upperTooth.pos.x) - int(lowerTooth.pos.x);
```

The following for - loop works pixel-wise along the contact line and calculates the damage. In a first step the position of opposing pixels expressed in i is calculated: It is i for the upper tooth and i_Lower for the lower tooth. Both differ by delta_pos_x.

The [YVALUE] dimension of the upper and the lower tooth keep the dimension of the teeth. In the beginning the [YVALUE] for every [i] is 0. When a pixel gets abrased, this is expressed by an increase of the [YVALUE]. By this the [YVALUE] defines the shape of the abrased teeth.

The [DAMAGE] dimension of both teeth keeps the damage of every pixel [i] alive.

In a first if-clause we check, if the specific pixel I of the upper tooth is in contact with the pixel i_Lower of the lower tooth. If so, in_touch is set to true and if the [DAMAGE] does not exceed the [STRUCTUR_PRESET] (i.e. the maximum damage in case of homogenous tooth hardness) the [DAMAGE] value for the specific pixels is increased by a random value between 0 and 2. If the [DAMAGE] exceeds the [STRUCTUR_PRESET], the [DAMAGE] is set to 0 again and the pixel gets abrased by increasing the [YVALUE]. This is done for the upper and lower arcade respectively.

```
for (int i =  startContactLine ; i < endContactLine; i++) {
i_Lower = i + delta_pos_x;
 if ((lowerTooth.pos.y-upperTooth.pos.y) <
     (HEIGHT_MOLAR - upperTooth.toothStructure[i][YVALUE]
       - lowerTooth.toothStructure[i_Lower][YVALUE])){
   in_touch = true;
    if (upperTooth.toothStructure[i][DAMAGE]
        < upperTooth.toothStructure[i][STRUCTUR_PRESET]){
      upperTooth.toothStructure[i][DAMAGE] += random(3); }
    else {
      upperTooth.toothStructure[i][YVALUE] += 1;
      upperTooth.toothStructure[i][DAMAGE] = 0;
    }
  if (lowerTooth.toothStructure[i_Lower][DAMAGE]
        < lowerTooth.toothStructure[i_Lower][STRUCTUR_PRESET]){
    lowerTooth.toothStructure[i_Lower][DAMAGE] +=  random(3); }
  else {
    lowerTooth.toothStructure[i_Lower][YVALUE] += 1;
    lowerTooth.toothStructure[i_Lower][DAMAGE] = 0;
    }
  }
}
```

If iterating through the i does not identify a touch the in_touch variable stays false and the next movement step of the arcade is one pixel upwards in dorsal direction. This is repeated, until a touch happens.

As a summary, the movement pattern of the lower cheek tooth is pixel wise dorsally until a touch happens. Then the [DAMAGE] and new structure [YVALUE] is calculated and with the next step of the tooth in buccal direction the yaw opens by 2 pixels in order to move dorsally again until the next touch happens.

```
      if (in_touch == false){
        lowerTooth.move.x =  0;
        lowerTooth.move.y = -1;

      }

    }
  lowerTooth.pos.add(lowerTooth.move);
```

Finally, the tooth position is changed by adding the move-vector to the current position.
